# Development of TASP0410457 (TASP457), a novel dihydroquinolinone derivative as a PET radioligand for central histamine H_3_ receptors

**DOI:** 10.1186/s13550-016-0170-2

**Published:** 2016-02-09

**Authors:** Kazumi Koga, Jun Maeda, Masaki Tokunaga, Masayuki Hanyu, Kazunori Kawamura, Mari Ohmichi, Toshio Nakamura, Yuji Nagai, Chie Seki, Yasuyuki Kimura, Takafumi Minamimoto, Ming-Rong Zhang, Toshimitsu Fukumura, Tetsuya Suhara, Makoto Higuchi

**Affiliations:** Molecular Imaging Center, National Institute of Radiological Sciences, 4-9-1 Anagawa, Inage-ku, Chiba, Chiba, 263-8555 Japan; Taisho Pharmaceutical Co., Ltd., 1-403 Yoshino-cho, Kita-ku, Saitama, 331-9530 Japan; Tohoku University Graduate School of Medicine, 2-1 Seiryo-cho, Aoba-ku, Sendai, 980-8575 Japan

**Keywords:** Positron emission tomography, Histamine H_3_ receptor, Receptor occupancy, Liquid chromatography and tandem mass spectrometry

## Abstract

**Background:**

Histamine H_3_ receptor (H_3_R) is a potential therapeutic target of sleep- and cognition-related disorders. The purpose of the present study is to develop a novel positron emission tomography (PET) ligand for H_3_Rs from dihydroquinolinone derivatives, which we previously found to have high affinity with these receptors.

**Methods:**

Six compounds were selected from a dihydroquinolinone compound library based on structural capability for ^11^C labeling and binding affinity for H_3_Rs. Their in vivo kinetics in the rat brain were examined in a comparative manner by liquid chromatography and tandem mass spectrometry (LC-MS/MS). Chemicals with appropriate kinetic properties were then labeled with ^11^C and evaluated in rats and monkeys using PET.

**Results:**

Of the six compounds, TASP0410457 (also diminutively called TASP457) and TASP0434988 exhibited fast kinetics and relatively high brain uptakes in ex vivo LC-MS/MS and were selected as candidate PET imaging agents. PET data in rat brains were mostly consistent with LC-MS/MS findings, and rat and monkey PET scans demonstrated that [^11^C]TASP0410457 was superior to [^11^C]TASP0434988 for high-contrast H_3_R PET imaging. In the monkey brain PET, distribution volume for [^11^C]TASP0410457 could be quantified, and receptor occupancy by a nonradioactive compound was measurable using this radioligand. The specific binding of [^11^C]TASP0410457 to H_3_Rs was confirmed by autoradiography using rat and monkey brain sections.

**Conclusions:**

We developed [^11^C]TASP0410457 as a radioligand enabling a robust quantification of H_3_Rs in all brain regions and demonstrated the utility of ex vivo LC-MS/MS and in vivo PET assays for selecting appropriate imaging tracers. [^11^C]TASP0410457 will help to examine the implication of H_3_Rs in neuropsychiatric disorders and to characterize emerging therapeutic agents targeting H_3_Rs.

**Electronic supplementary material:**

The online version of this article (doi:10.1186/s13550-016-0170-2) contains supplementary material, which is available to authorized users.

## Background

Histaminergic neurotransmission in the brain appears to play roles in drinking and feeding, learning and memory, and wake and sleep [1, 2]. Histamine H_3_ receptor (H_3_R) acts as either presynaptic autoreceptor or heteroreceptor, producing negative feedback regulation of histamine release or regulating release of non-histamine neurotransmitters including glutamate, norepinephrine, dopamine, and serotonin [3–6]. Therefore, H_3_R can be a potential therapeutic target for various diseases such as sleep-wake disorders including narcolepsy, attention-deficit hyperactivity disorder, Alzheimer’s disease, and schizophrenia [7–9].

Positron emission tomography (PET) imaging techniques offer analytical approaches for examining neuroreceptor systems. Neuroreceptor ligands applicable to PET imaging could provide tools to investigate alterations in specific neurotransmissions under pathological conditions and to quantify the receptor occupancy by therapeutic agents. However, developing PET ligands for new targets has often been challenging, as an appropriate PET ligand needs to satisfy several criteria including the following properties: sufficient binding affinity and selectivity for the target receptor, adequate lipophilicity and molecular size for blood–brain barrier penetration, minimal interference of radiometabolites with imaging assays, low nonspecific binding, presence of a radiolabeling sites, and sufficiently fast kinetics in the brain [10].

Two compounds have been used to image central H_3_Rs in clinical PET studies. GSK189254, a benazepin derivative, is an inverse H_3_R agonist developed to improve cognitive functions in Alzheimer’s disease [5] and was labeled with ^11^C for PET imaging of H_3_Rs. [^11^C]GSK189254 could visualize H_3_Rs in the human brains, but its slow kinetics impeded accurate quantification of the available binding sites in brain regions with high-level H_3_R expression [11]. MK-8278, a spiro-isobenzofuranone derivative, and an inverse agonist with high potency to H_3_Rs as well [12], was also labeled with ^11^C. [^11^C]MK-8278 showed more favorable kinetics than [^11^C]GSK189254 [13], although it still remains to be established whether the specific binding of this radioligand reaches an equilibrium state within the PET image acquisition time. Although these ligands were used for clinical trials to quantitatively estimate occupancy of H_3_Rs [11, 13–15], an additional new PET ligand with appropriate pharmacokinetic, metabolic, and binding characteristics can be developed along with a suitable method for quantitative determinations of kinetic parameters.

Ex vivo assays of an exogenously administered compound in tissues by liquid chromatography and tandem mass spectrometry (LC-MS/MS) have been used to evaluate kinetics of compounds in the brain without radiolabeling and to predict the capability of compounds as PET radioligands for several target molecules, such as opioid κ receptor and nociception/orphanin FQ receptor [16–20]. With this method, it is possible to select candidate compounds with preferable in vivo kinetics with a relatively high throughput. The recent advances of the LC-MS/MS device have also offered highly sensitive measurements of compound retaining in the brain following administration at a dose nearly equivalent to PET studies (approximately 2 to 10 μg/kg).

Here, we developed a novel PET ligand for H_3_Rs from dihydroquinolinone derivatives, which were shown to exert high affinity for H_3_Rs in our previous work [21]. Six compounds were initially selected from a library of dihydroquinolinone analogs in consideration of the presence of ^11^C labeling site and binding reactivity with H_3_Rs. Then, the pharmacokinetics of these candidate compounds in the rat brain were compared by ex vivo LC-MS/MS assays. Based on these kinetic data, two compounds were chosen for labeling with ^11^C and were evaluated in rats and monkeys using PET. A compound that exhibited more favorable kinetics and higher imaging contrast for H_3_Rs in the brain was considered as the best radioligand, and its utility in receptor occupancy studies was further demonstrated by PET in monkeys.

## Methods

### Chemicals

**Fig. 1:**
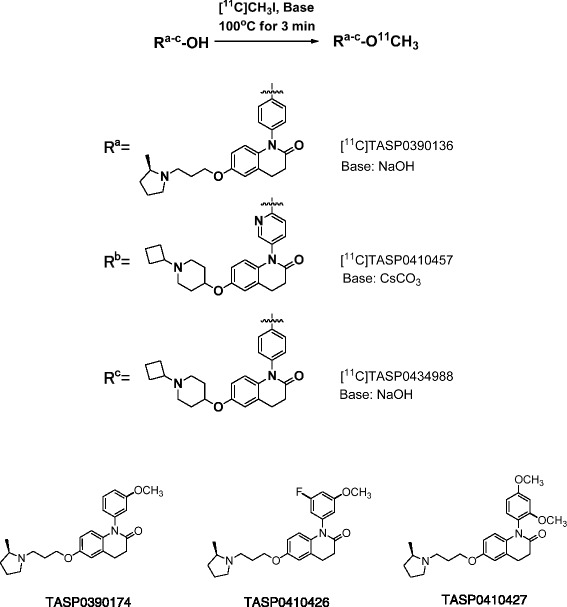
Radiosynthesis of [^11^C]TASP0390136, [^11^C]TASP0410457, and [^11^C]TASP0434988, and chemical structure of TASP0390174, TASP0410426, and TASP0410427

Dihydroquinolinone derivatives (Fig. 1), 1-(4-methoxyphenyl)-6-{3-[(2*R*)-2-methylpyrrolidin-1-yl]propoxy}-3,4-dihydroquinolin-2(1*H*)-one (TASP0390136), 6-[(1-Cyclobutylpiperidin-4-yl)oxy]-1-(6-methoxypyridin-3-yl)-3,4-dihydroquinolin-2(1*H*)-one (TASP0410457), 6-[(1-cyclobutylpiperidin-4-yl)oxy]-1-(4-methoxyphenyl)-3,4-dihydroquinolin-2(1*H*)-one (TASP0434988), 1-(3-methoxyphenyl)-6-{3-[(2*R*)-2-methylpyrrolidin-1-yl]propoxy}-3,4-dihydroquinolin-2(1*H*)-one (TASP0390174), 1-(3-fluoro-5-methoxyphenyl)-6-{3-[(2*R*)-2-methylpyrrolidin-1-yl]propoxy}-3,4-dihydroquinolin-2(1*H*)-one (TASP0410426), and 1-(2,4-dimethoxyphenyl)-6-{3-[(2*R*)-2-methylpyrrolidin-1-yl]propoxy}-3,4-dihydroquinolin-2(1*H*)-one (TASP0410427) were synthesized at Taisho Research Laboratories (Saitama, Japan). Procedures for synthesizing TASP0390136, TASP0410457, and TASP0434988 and precursors for ^11^C-labeling of these compounds are provided in Additional file 1: Supplemental methods. Thioperamide maleate and ciproxifan hydrochloride were purchased from Sigma-Aldrich (St. Louis, MO). Clobenpropit was purchased from Tocris Bioscience (Bristol, UK).

### In vitro binding of compounds to H_3_Rs

Rat cerebral cortex and monkey frontal cortex were dissected, homogenized with 50 mM Tris buffer (pH 7.4) containing 5 mM EDTA and Protease Inhibitor Cocktail Tablets (Roche Diagnostics, Indianapolis, IN) and centrifuged at 48,000×*g* for 15 min at 4 °C. The pellets were then suspended in binding buffer (50 mM Tris containing 5 mM EDTA, pH 7.4) and stored at −80 °C as membrane preparations.

The membrane preparations were thawed and incubated in the binding buffer containing 0.1 % bovine serum albumin, 1 nM [^3^H]N-α-methylhistamine (PerkinElmer, Waltham, MA) with various concentrations of a dihydroquinolinone derivative (i.e., test compound) at 25 °C for 1 h. The reaction was terminated by rapid filtration through 96-well GF/C filter plates (PerkinElmer) presoaked with 0.5 % polyethyleneimine (Wako, Osaka, Japan). After washes with ice-cold binding buffer, the plates were dried and filled with Microscint-o (PerkinElmer). The radioactivity retained on the filters was counted using a TopCount NXT (PerkinElmer). Total and nonspecific radioligand binding was determined by reaction without and with 10 μM thioperamide and clobenpropit, respectively. The specific radioligand binding was then calculated by subtracting the nonspecific binding from the total binding. The IC_50_ value for inhibition of the radioligand binding by the test compound was determined using a nonlinear fit of the concentration-response curve for each test compound using SAS 9.2 (SAS Institute Inc., Cary, NC). Data were expressed as the geometric means of IC_50_ values from three independent experiments run in duplicate.

### Other in vitro profiling of compounds

Lipophilicity, off-target binding, and P-glycoprotein-mediated efflux were determined as described in Additional file 1: Supplemental methods.

### Ex vivo pharmacokinetic assessments of compounds in the rat brain using LC-MS/MS

The procedures of this animal experiment were reviewed and approved by the Animal Care Committee of Taisho Pharmaceutical Co., Ltd. Six-week-old male Sprague-Dawley rats were purchased from Charles River (Yokohama, Kanagawa, Japan).

The rats were decapitated at 5, 15, 30, 60, and 120 min after intravenous administration of TASP0390136, TASP0390174, TASP0410426, TASP0410427, TASP0410457, or TASP0434988 at a dose of 10 μg/kg. The cerebellum and forebrain, including the medial prefrontal and cingulate cortices and anterior striatum, were dissected and homogenized with four volumes of distilled water. The homogenates were protein-precipitated with methanol/acetonitrile (1:9) and were centrifuged at 3974×*g* for 10 min at 4 °C. The resultant supernatants were injected into an HPLC (Shimadzu-20AD, Shimadzu, Kyoto, Japan) and a tandem mass spectrometer (API4000, AB SCIEX, Framingham, MA) using a Shim-pak XR-ODS column (3.0 × 30 mm, Shimadzu) at 50 °C. TASP0410457 was eluted with a mobile phase consisting of 10 mM of ammonium acetate/acetonitrile with a linear gradient at a flow rate of 1.3 mL/min, and ammonium formate instead of ammonium acetate was used for eluting the other five compounds. This modification was effective only for assaying TASP0410457, resulting in the reduction of the low limit of quantification but did not improve the detection of other five compounds. The eluted TASP0390136, TASP0390174, TASP0410426, TASP0410427, TASP0410457, and TASP0434988 were ionized using an electrospray interface and were detected using reaction monitoring of the transitions of m/z 395 to 91, 395 to 98, 413 to 98, 425 to 98, 408 to 138, and 407 to 138, respectively. A dihydroquinolinone compound (6-[(1-cyclobutylpiperidin-4-yl)oxy]-1-(3,5-difluorophenyl)-3,4-dihydroquinolin-2(*1H*)-one) was used as an internal standard for all assays. The lower qualification limit for TASP0410457 was 0.05 ng/g tissue, and the limit for the other five compounds was 0.15 ng/g tissue. Data were expressed as standardized uptake values (SUVs) calculated as follows:

$$ \mathrm{S}\mathrm{U}\mathrm{V}\kern0.5em =\kern0.5em \frac{\mathrm{tissue}\kern0.5em \mathrm{concentration}\kern0.5em \left(\mathrm{g}/\mathrm{g}\right)\times \mathrm{body}\kern0.5em \mathrm{weight}\kern0.5em \left(\mathrm{g}\right)}{\mathrm{injected}\kern0.5em \mathrm{dose}\kern0.5em \left(\mathrm{g}\right)} $$

### Radiochemical synthesis

[^11^C]TASP0390136, [^11^C]TASP0410457, and [^11^C]TASP0434988 were synthesized as shown in Fig. 1a–c (detailed procedures are described in Additional file 1: Supplemental methods) using a homemade automated synthesis system [22]. The radiochemical purities and specific activities of the radioligands, which were analyzed as described in Additional file 1: Supplemental Methods, along with their radiochemical yields and average synthesis times, are summarized in Table 1. Analytical HPLC chromatogram of each radioligand is shown in Additional file 2-4: Figures S1-S3. The radiochemical stabilities of [^11^C]TASP0410457 and [^11^C]TASP0434988 in the final formulation were >99 % at 1 h and 40 min, respectively, after the end of radiosynthesis.

**Table 1:** Summary of radiosynthesis

	Radiochemical yield^ab^ (%)	Average synthesis time (min)	Specific activity^ac^ (GBq/mol)	Radiochemical purity^c^ (%)
[^11^C]TASP0390136	10.1 ± 3.1	29	95 ± 23	>98
[^11^C]TASP0410457	1.7 ± 0.3	29	104 ± 7	>98
[^11^C]TASP0434988	15.5 ± 4.6	29	111 ± 16	>98

^a^Mean ± S.E.M., *n* = 3–8

^b^Based on [^11^C]CO_2_ obtained by bombardment with decay-uncorrected at the end of synthesis (EOS)

^c^Data at EOS

### PET imaging of rat brains

All PET experiments using animals were approved by the Committee for the Care and Use of Laboratory Animals of the National Institute of Radiological Sciences (NIRS).

Six-week-old male Sprague-Dawley rats were purchased from Japan SLC (Shizuoka, Japan). PET scans were performed using a microPET Focus 220 small-animal scanner (Siemens Medical Solutions USA, Knoxville, TN) [23]. The rats were anesthetized with 1.5–2 % isoflurane during the imaging session, while their heart rate and blood oxygen saturation were monitored by pulse oximeter (CANL-425SVA; Med Associates, St. Albans, VT). Body temperature was kept at 37 °C with a heating pad (BWT-100; Bio Research Center, Aichi, Japan). Following the transmission scans for attenuation correction using a ^68^Ge-^68^Ga point source, emission scans were performed for 90 min in 3D list-mode with an energy window of 350–750 keV, immediately after the intravenous injection of each radioligand. Mean ± S.D. of the injected radioactivity of [^11^C]TASP0410457, [^11^C]TASP0434988, and [^11^C]TASP0390136 was 119 ± 26 MBq, 132 ± 34 MBq, and 124 ± 17 MBq, respectively (*n* = 3–5). Mean ± S.D. of the mass doses of [^11^C]TASP0410457, [^11^C]TASP0434988, and [^11^C]TASP0390136 was 2.4 ± 1.2 μg/kg, 1.9 ± 0.4 μg/kg, and 4.2 ± 1.6 μg/kg, respectively (*n* = 3–5).

To assess the specificity of radioligand binding, thioperamide was injected intravenously at a dose of 10 mg/kg at 30 s before the injection of each radioligand.

### Analyses of rat PET data

All list-mode data were sorted into 3D sinograms, which were then Fourier rebinned into 2D sinograms (frames: 1 min × 4, 2 min × 8 and 5 min × 14). Images were reconstructed using 2D-filtered back-projection with a 0.5-mm Hanning filter.

PET image and magnetic resonance imaging (MRI) template were co-registered using PMOD software (PMOD version 3.206; Zurich, Switzerland) according to the following procedure as in our previous works: Outline of the whole brain and positions of eyes in PET images at an early phase of the scan were initially matched to those in MRI images, and then further translations and rotations of PET images were manually performed for fine alignments. Finally, parameters for the co-registration were applied to the entire dynamic PET data. Regions of interest (ROIs) were placed on the striatum, hippocampus, and cerebellum using PMOD software with reference to the MRI template as described elsewhere [24]. The tissue time-activity curves (TACs) for these ROIs were generated by calculating the SUV for each time frame as follows:

$$ \mathrm{S}\mathrm{U}\mathrm{V}=\frac{\mathrm{radioactivity}\kern0.5em \left(\mathrm{Bq}/\mathrm{g}\right) \times \mathrm{body}\kern0.5em \mathrm{weight}\kern0.5em \left(\mathrm{g}\right)}{\mathrm{injected}\kern0.5em \mathrm{radiotracer}\kern0.5em \mathrm{dose}\kern0.5em \left(\mathrm{Bq}\right)} $$

The binding potentials relative to the non-displaceable uptake (BP_ND_) in the striatum and hippocampus were estimated using the simplified reference tissue model (SRTM) [25] with PMOD software using the cerebellum as a reference region, as the rat cerebellum is devoid of H_3_R [26].

### PET study in monkeys

A male rhesus monkey (*Macaca mulatta*, weighing 6–7 kg) was used for a comparative PET analysis of [^11^C]TASP0410457 and [^11^C]TASP0434988. PET scans were performed using a high-resolution SHR-7700 animal PET camera (Hamamatsu Photonics, Hamamatsu, Japan). Following transmission scans for attenuation correction using a ^68^Ge-^68^Ga point source, emission scans were performed on a conscious monkey as described previously [27], in 3D acquisition mode (frames: 10 s × 12, 30 s × 6, 1 min × 5, 2 min × 5 and 5 min × 14) for 90 min after the intravenous injection of [^11^C]TASP0410457 (169–176 MBq) or [^11^C]TASP0434988 (173–188 MBq). Briefly, the monkey was immobilized by joining the acrylic cap on the monkey head with the fixation device during PET scans. To assess a homologous competition for the target binding sites, unlabeled TASP0410457 or TASP0434988 was injected intravenously at a dose of 1 mg/kg at 30 s before injection of the corresponding radioligand.

For a detailed pharmacokinetic PET study using [^11^C]TASP0410457 with arterial blood sampling, three male rhesus monkeys weighing 5–7 kg were scanned with the SHR-7700 PET camera. The monkeys were initially anesthetized with an intramuscular injection of ketamine (10 mg/kg), intubated, and subsequently kept anesthetized with 1–2 % isoflurane. Electrocardiogram and blood oxygen saturation levels were monitored, and body temperature was maintained by a heating pad throughout the experiment. Arterial blood (1–2.5 mL) was collected from the saphenous artery at 10, 20, 30, 40, 50, 60, and 90 s and at 2, 3, 4, 5, 10, 15, 30, 60, and 90 min after the intravenous injection of [^11^C]TASP0410457 (injected radioactivity, 159 ± 30 MBq; mass dose, 1.2 ± 0.3 μg; mean ± S.D., *n* = 3).

The arterial blood was centrifuged at 20,000×*g* for 3 min at 4 °C to separate the plasma. Radioactivity in total blood and plasma was determined using a 1480 WIZARD gamma counter (PerkinElmer), and was decay-corrected to the injection time. Arterial plasma collected at 1, 5, 15, 30, 60, and 90 min was used to analyze radioactive metabolites of [^11^C]TASP0410457. Two hundred microliters of plasma was mixed with 200 μL of ice-cold acetonitrile, and the mixtures were centrifuged at 20,000×*g* for 3 min at 4 °C. Fifty to five hundred microliters of the resultant supernatants were separated using HPLC (JASCO, Tokyo, Japan) on an Atlantis T3 column (4.6 × 50 mm, Waters, Milford, MA) with a mobile phase consisting of 10 mM of ammonium acetate/acetonitrile (68:32) at a flow rate of 1 mL/min. The fraction of unmetabolized [^11^C]TASP0410457 was determined by measuring the areas of the HPLC peaks corresponding to [^11^C]TASP0410457 and its radiometabolites.

### Analyses of monkey PET data

Emission scan images were reconstructed using a 4.0-mm Colsher filter. ROIs were placed on the anterior cingulate cortex, striatum, thalamus, hippocampus, cerebellum, frontal cortex, temporal cortex, occipital cortex, and pons-medulla using PMOD software, with reference to an MRI template as described previously [24]. Tissue TACs were generated as in the rat PET image analysis.

For PET study in anesthetized monkeys, metabolite-corrected plasma TACs were used as arterial input functions. The regional total volume of distribution (*V*_T_) was estimated by Logan’s graphical plot and one-tissue compartment model using PMOD software.

### Receptor occupancy assays

Occupancy of central H_3_Rs by ciproxifan, a selective antagonist/inverse agonist, was quantified by [^11^C]TASP0410457-PET scans of an anesthetized monkey with arterial blood sampling. Ciproxifan was injected at a dose of 3 mg/kg intravenously at 10 min before the injection of [^11^C]TASP0410457. Receptor occupancy of ciproxifan was determined using Lassen’s plot without any reference regions using the following equation:

$$ {V_{\mathrm{T}}}^{\mathrm{Baseline}}-{V_{\mathrm{T}}}^{\mathrm{Ciproxifan}}=\mathrm{O}\mathrm{c}\mathrm{c}\left({V_{\mathrm{T}}}^{\mathrm{Baseline}}-{V}_{\mathrm{ND}}\right) $$

where *V*_T_^Baseline^ and *V*_T_^Ciproxifan^ are the regional *V*_T_ values at baseline and in the ciproxifan challenge, respectively, *V*_ND_ is the non-displaceable distribution volume, and Occ is the occupancy [28].

### Autoradiography of [^11^C]TASP0410457 using rat and monkey brain sections

Twenty-micrometer-thick sagittal rat and monkey brain sections were incubated in an autoradiography buffer containing 5 nM [^11^C]TASP0410457 as described in Additional file 1: Supplemental methods. The binding was also performed in the presence of 10 μM of unlabeled TASP0410457, thioperamide, or ciproxifan for the rat sections and 10 μM of TASP0410457 or ciproxifan for the monkey sections to assess the specific radioligand binding.

## Results

### Selection of candidate compounds for PET imaging

**Fig. 2:**
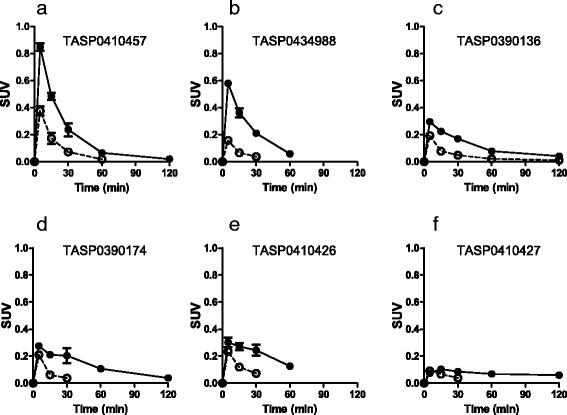
Pharmacokinetics of non-radiolabelled compounds in the rat brain. **a**–**f** The concentrations of TASP0410457 (**a**), TASP0434988 (**b**), TASP0390136 (**c**), TASP0390174 (**d**), TASP0410426 (**e**), and TASP0410427 (**f**) in the forebrain (*closed circles*) and cerebellum (*open circles*) were determined by ex vivo LC-MS/MS after the intravenous administration of 10 μg/kg of the compound in rats. The absence of symbols at 60 or 120 min in the plots indicates that the concentration measures were below the lower limit of quantification. Data are mean ± S.E.M (*n* = 3)

Six compounds, TASP0410457, TASP0434988, TASP0390136, TASP0390174, TASP0410426, and TASP0410427, were initially selected from our dihydroquinolinone compound library, since they fulfilled the following criteria for a PET radioligand candidate: (1) the presence of a methoxy group or other moieties for ^11^C or ^18^F labeling and (2) high binding affinity for rat H_3_R (IC_50_ < 3 nM). The kinetics of these compounds in the rat brain were then pursued by ex vivo LC-MS/MS measurements of non-labeled compounds in brain tissues collected at different time points after bolus intravenous administration (Fig. 2). The ranked order of the peak concentration of these compounds in the forebrain including striatum enriched with H_3_Rs was TASP0410457 > TASP0434988 > TASP0390136 ≈ TASP0390174 ≈ TASP0410426 > TASP0410427 (Fig. 2). Mean peak concentrations of TASP0410457, TASP0434988, TASP0390136, TASP0390174, TASP0410426, and TASP0410427 in the forebrain expressed as SUV were 0.85, 0.58, 0.30, 0.28, 0.30, and 0.10, respectively (Fig. 2). The concentrations of all six compounds in the forebrain were higher than those in the cerebellum devoid of H_3_Rs throughout the pharmacokinetic tracking.

TASP0410457 and TASP0434988 were selected as candidates for a PET radioligand, because they exhibited rapid entry into the brain followed by fast washout (Fig. 2a, b) and the highest and second highest uptakes, respectively, in the forebrain versus the cerebellum. TASP0390136 was also chosen for radiolabeling as a control compound with insufficient brain uptake in order to assess the consistency between ex vivo LC-MS/MS and in vivo PET data.

We then revisited the in vitro properties of these three compounds in search of chemical determinants of in vivo pharmacokinetic performance of test ligands in the brain. The three compounds showed high in vitro binding affinity for rat and monkey H_3_Rs (Table 2) and displayed more than 60 times higher selectivity for H_3_R than off-target binding components including sigma 1, adrenergic α_2c_ receptors, and 67 other molecules (Additional file 1: Table S1). Lipophilicity of these chemicals was slightly low to moderate as CNS-targeting compounds (Table 2). Based on the susceptibility for P-glycoprotein with an assumed criterion for efflux ratio (ER) exceeding 2.00, TASP0410457 was not a substrate of P-glycoprotein (ER: 1.64), while TASP0390136 and TASP0434988 turned out to be P-glycoprotein substrates (ER: 6.29 and 2.14, respectively). It should be noted that the ex vivo LC-MS/MS measures were only partly correlated with the susceptibility of the compounds for human P-glycoprotein. Indeed, uptakes of TASP0390136 and TASP0434988 in the cerebellum were similar despite a large difference in the ER estimate (Fig. 2b, c). Hence, diverse factors determining the permeability of chemicals into the brain, including species difference in P-glycoprotein and contributions other efflux transporters, should also be taken into account.

**Table 2:** In vitro properties of TASP0390136, TASP0410457, and TASP0434988

	Affinity for H_3_Rs		Off-target binding		Log D_7.4_	Efflux ratio (MDR1)
	Rat, IC_50_	Monkey, IC_50_	Sigma 1, IC_50_	Adrenergic α_2c_, IC_50_		
	nM (*n* = 3)	nM (*n* = 3)	nM	nM		
TASP0390136	2.74	1.06	167	>1000	1.36	6.29
TASP0410457	2.34	1.80	379	>1000	1.79	1.64
TASP0434988	0.783	0.565	257	528	2.22	2.14

### PET imaging of the rat brain with [^11^C]TASP0410457 and [^11^C]TASP0434988 versus [^11^C]TASP0390136

**Fig. 3:**
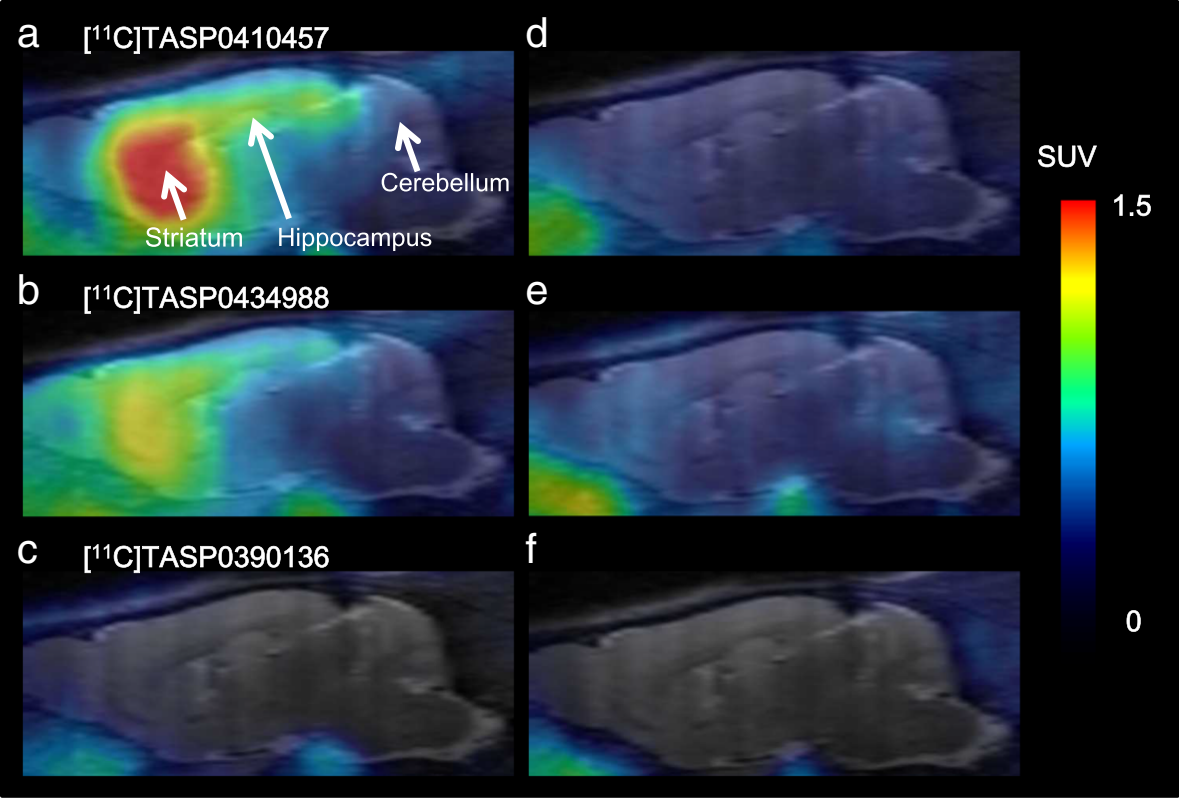
In vivo PET images of [^11^C]TASP0410457, [^11^C]TASP0434988, and [^11^C]TASP0390136 in the rat brain. Average sagittal PET images at 0–90 min after intravenous administration of [^11^C]TASP0410457 (*top*), [^11^C]TASP0434988 (*middle*), and [^11^C]TASP0390136 (*bottom*) at baseline (**a**–**c**) and after pretreatment with 10 mg/kg of thioperamide (**d**–**f**). The PET images were overlaid on an MRI template

**Fig. 4:**
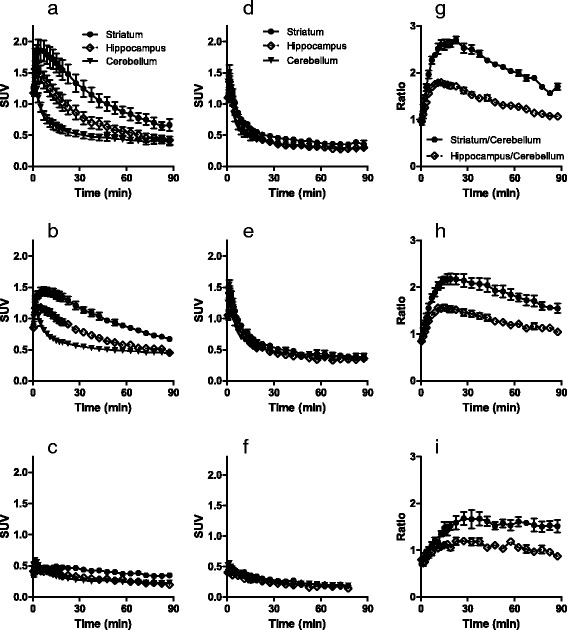
**a**–**f**: TACs for [^11^C]TASP0410457 (*top*), [^11^C]TASP0434988 (*middle*), and [^11^C]TASP0390136 (*bottom*) in the rat striatum (*circles*), hippocampus (*rhombi*), and cerebellum (*triangles*) at baseline (**a**–**c**) and after pretreatment with 10 mg/kg of thioperamide (**d**–**f**). **g**–**i** Temporal changes of specifically bound [^11^C]TASP0410457 (**g**), [^11^C]TASP0434988 (**h**), and [^11^C]TASP0390136 (**i**) in the striatum (*circles*) and hippocampus (*rhombi*) determined as ratios in SUVs between the target region and the cerebellum. Data are mean ± S.E.M (*n* = 3–5)

PET scans of rats demonstrated high radioactivity signals in the striatum after a bolus intravenous administration of [^11^C]TASP0410457 (Fig. 3a) with a peak SUV approximating 2.0, and the hippocampus and cerebellum exhibited moderate and low signals with SUVs peaking at around 1.5 and 1.2, respectively (Fig. 4a), in agreement with the known distribution of H_3_R. Although the peak uptake of [^11^C]TASP0434988 in the cerebellum was only slightly lower than that of [^11^C]TASP0410457, striatal and hippocampal peak SUVs for [^11^C]TASP0434988 were 20–25 % lower than those for [^11^C]TASP0410457 (Figs. 3b, 4b). [^11^C]TASP0390136 yielded profoundly lower signals in all brain regions than the other two radioligands (Figs. 3c, 4c), in accordance with ex vivo LC-MS/MS observations.

The regional differences in radioactivity were abolished by the pretreatment with 10 mg/kg of thioperamide, an H_3_R antagonist (Figs. 3d–f, 4d–f), or 1 mg/kg of unlabeled corresponding compounds (data not shown). The cerebellar kinetics of each radioligand was not noticeably altered by these pretreatments (Fig. 4a–f), indicating the availability of the cerebellum as a reference region for quantitative analyses in rats. Indeed, cerebellar SUVs at 15 min after radioligand injection at baseline and thioperamide predosing were 0.64 ± 0.13 and 0.47 ± 0.09, respectively, for [^11^C]TASP0410457, 0.64 ± 0.04 and 0.59 ± 0.02, respectively, for [^11^C]TASP0434988, and 0.33 ± 0.01 and 0.30 ± 0.03, respectively, for [^11^C]TASP0390136 (mean ± S.D.). Target-to-reference ratios of the radioligand retention indicated that the specific binding of [^11^C]TASP0410457 and [^11^C]TASP0434988 reached a pseudo-equilibrium state within 15 min after intravenous injection (Fig. 4g, h). The specific binding of [^11^C]TASP0410457 determined as BP_ND_ was 25–30 % higher than that of [^11^C]TASP0434988 in the striatum and hippocampus (Table 3).

**Table 3:** Regional BP_ND_ values for radioligands in rat PET

	Striatum	Hippocampus
[^11^C]TASP0410457	1.26 ± 0.12	0.53 ± 0.06
[^11^C]TASP0434988	0.92 ± 0.21	0.36 ± 0.11

The regional brain kinetics of the three compounds exhibited by PET was mostly consistent with the ex vivo LC-MS/MS results. The ranked order of peak radioligand uptakes in the striatum agreed with the order in the forebrain determined by LC-MS/MS (TASP0410457 > TASP0434988 > TASP0390136; Fig. 2a–c versus Fig. 4a–c). Meanwhile, the cerebellar peak radioligand uptakes assayed by PET but not ex vivo LC-MS/MS were inversely correlated with the ER values of the compounds. As a consequence, the LC-MS/MS analysis seemed to overestimate the specific binding (difference in SUV between the target reference regions) of TASP0434988 as compared with the quantification by PET.

### Comparative PET imaging of monkey brains with [^11^C]TASP0410457 and [^11^C]TASP0434988

**Fig. 5:**
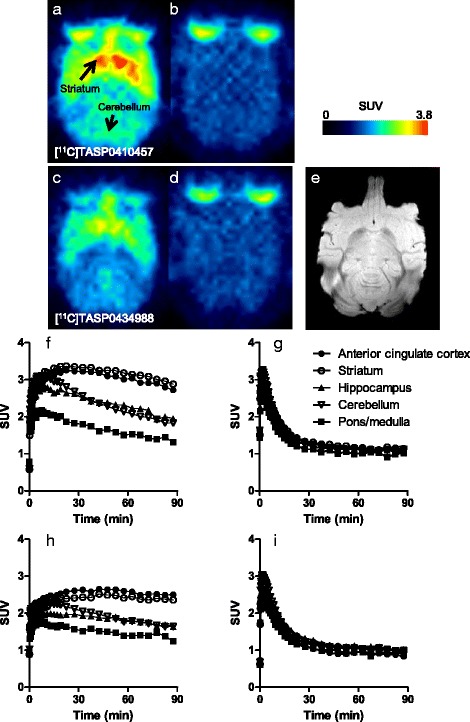
In vivo PET in a conscious rhesus monkey. **a**–**e** Average horizontal PET images at 30–90 min after the intravenous administration of [^11^C]TASP0410457 (*top*) and [^11^C]TASP0434988 (*bottom*) at baseline (**a**, **c**) and after pretreatment with 1 mg/kg of unlabelled TASP0410457 (**b**) or TASP0434988 (**d**), along with co-registered MR images of the monkey brain (**e**). **f**–**i** TACs for [^11^C]TASP0410457 (*top*) and [^11^C]TASP0434988 (*bottom*) in the anterior cingulate cortex (*closed circles*), striatum (*open circles*), hippocampus (*closed triangles*), cerebellum (*open triangles*), and pons-medulla (*squares*) at baseline (*left*) and after pretreatment with 1 mg/kg of unlabelled compound (*left*)

Radioactivity signals in the monkey striatum and anterior cingulate cortex were intensified after the bolus intravenous administration of [^11^C]TASP0410457 and [^11^C]TASP0434988 (Fig. 5a, c). The uptake of [^11^C]TASP0410457 peaked within 30 min after the injection, followed by a gradual decline (Fig. 5f), and [^11^C]TASP0434988 underwent a slightly slower washout from the brain than [^11^C]TASP0410457 (Fig. 5h). Radioactivity in all ROIs, including the cerebellum and pons/medulla, was decreased by pretreatment with unlabeled corresponding compounds (Fig. 5b, d, g, and i), indicating that the radioligand binding was saturable and specific and that no brain areas in the monkey were available as reference regions for quantifications. The difference in regional radioligand retention beyond 30 min between baseline and blocking studies was greater in [^11^C]TASP0410457-PET than in [^11^C]TASP0434988-PET (Fig. 5), supporting the advantage of [^11^C]TASP0410457 over [^11^C]TASP0434988 for high-contrast imaging of central H_3_Rs. We accordingly chose [^11^C]TASP0410457 for further characterizations as a radioligand with the highest in vivo performance.

### Quantitative analyses of [^11^C]TASP0410457-PET data in monkeys

**Fig. 6:**
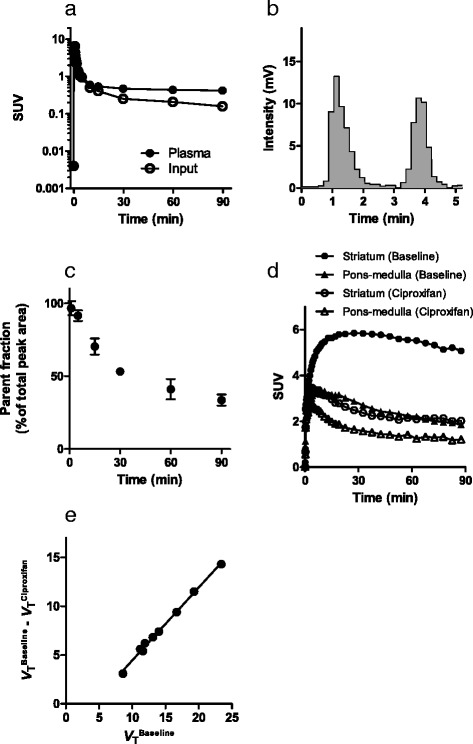
In vivo PET in anesthetized rhesus monkeys with acquisition of an arterial input function. **a**–**c** Representative plasma TAC (*closed circles*) and metabolite-corrected arterial input function (*open circles*) in a monkey (**a**), a representative radio-HPLC chromatogram of the protein-precipitated monkey plasma at 30 min after an administration of [^11^C]TASP0410457 (**b**) and time course of changes in the ratio of unmetabolized [^11^C]TASP0410457 in plasma (**c**; mean ± S.E.M, *n* = 3). **d** TACs in the striatum (*circles*) and pons-medulla (*triangles*) at baseline (*closed symbols*) and after pretreatment with 3 mg/kg of ciproxifan (*open symbols*) obtained in a single monkey. **e** Lassen plot for determination of the occupancy of H_3_Rs by ciproxifan. A *circle* in the Lassen plot denotes a value in each ROI, and a *solid line* represents regression

The uptake of [^11^C]TASP0410457 in the brain was quantified with arterial blood sampling in anesthetized monkeys. Plasma radioactivity rapidly increased after the injection, peaked at approximately 1 min, and then promptly declined (Fig. 6a). A reverse-phase HPLC analysis of the monkey plasma indicated that a major metabolite of [^11^C]TASP0410457 (the first peak in Fig. 6b) with a retention time corresponding to the holdup time was more polar than the parent compound (the second peak in Fig. 6b). The fraction of unmetabolized [^11^C]TASP0410457 decreased over time, accounting for 34 % of total plasma radioactivity at 90 min (Fig. 6c). Radioligand retention was increased particularly in H_3_R-rich brain areas of these anesthetized monkeys (Fig. 6d) relative to a conscious monkey (Fig. 5f), implying anesthetic-induced enhancement of radioligand binding. To examine this possibility, we carried out an additional PET study using another monkey in a conscious condition. The peak SUV in the striatum of this monkey was 3.0 and was similar to the value (3.4) in the first conscious monkey. These values were below mean–2S.D. in anesthetized monkeys (5.0 ± 0.8, mean ± S.D.), further supporting effects of anesthesia. However, radioligand retention peaked at around 30 min in the striatum of anesthetized monkeys, which was similar to the kinetics in the awake monkey. A time-stability examination demonstrated that the progressive truncation of the PET data had relatively small but noticeable effects (within 7 % of the values for 90 min) on the *V*_T_ values estimated by Logan’s graphical analysis when the endpoint of the sampling time interval was changed from 90 to 60 min (Table 4). We additionally calculated *V*_T_ values by a one-compartment model and obtained estimates similar to the graphical plot results (Table 4).

**Table 4:** Time-stability of regional *V*_T_ values for [^11^C]TASP0410457 in the monkey brain

	Logan graphical analysis				One-tissue compartment model
	Scan duration (min)				Scan duration (min)
Region	60	70	80	90	90
Striatum	21.2 ± 3.0	21.5 ± 3.0	21.9 ± 3.3	22.6 ± 3.8	23.2 ± 3.6
Anterior cingulate cortex	18.4 ± 2.7	18.6 ± 2.8	19.1 ± 3.2	19.5 ± 3.4	20.3 ± 3.3
Thalamus	15.1 ± 1.7	15.4 ± 1.9	15.6 ± 1.9	16.0 ± 2.1	16.1 ± 1.8
Hippocampus	13.0 ± 1.0	13.2 ± 1.0	13.6 ± 1.2	13.9 ± 1.4	13.9 ± 1.3
Frontal cortex	12.3 ± 1.6	12.5 ± 1.7	12.8 ± 1.8	13.0 ± 2.0	13.4 ± 1.8
Cerebellum	12.1 ± 1.2	12.4 ± 1.3	12.7 ± 1.5	13.0 ± 1.7	12.8 ± 1.7
Temporal cortex	12.3 ± 1.6	12.4 ± 1.7	12.5 ± 1.8	12.9 ± 2.1	13.3 ± 2.1
Occipital cortex	11.7 ± 1.6	11.8 ± 1.6	12.0 ± 1.8	12.1 ± 1.9	12.7 ± 2.0
Pons-medulla	8.5 ± 0.6	8.6 ± 0.7	8.8 ± 0.7	9.1 ± 0.9	8.8 ± 0.9

Mean ± S.D., *n* = 3

Pretreatment by intravenous administration of an H_3_R antagonist, ciproxifan, at a dose of 3 mg/kg substantially reduced the radioactivity in all ROIs including the cerebellum and pons-medulla. Lassen’s plot displayed robust linearity (Fig. 6e), demonstrating uniform receptor occupancy among brain regions, and the receptor occupancy of ciproxifan at this dose was stably estimated as ~75 %. Non-displaceable distribution volume was estimated as 4.0 mL/cm^3^ (Fig. 6e), and thus, the specific radioligand binding accounted for more than 80 % of the radioactivity in the striatum, taking this result together with the finding that the striatal *V*_T_ exceeded 20 mL/cm^3^ (Table 4). We also treated a monkey with 10 mg/kg of ciproxifan but could not complete PET imaging due to its profound effects on heart rates and body motions even in an anesthetized condition with isoflurane.

### Autoradiography of [^11^C]TASP0410457

**Fig. 7:**
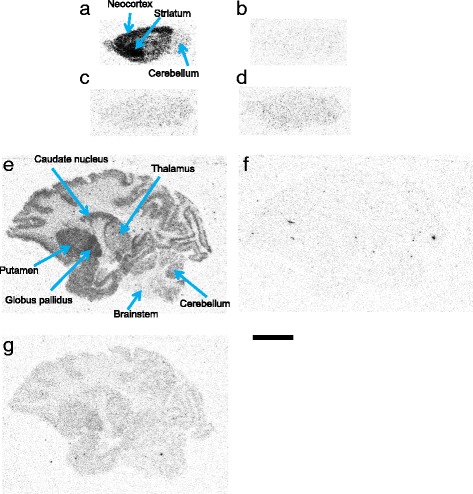
In vitro autoradiographic labeling of rat and rhesus monkey brain slices with [^11^C]TASP0410457. The images show radioligand bindings in sagittal rat brain sections without blockade (**a**) or with 10 μM of unlabelled TASP0410457 (**b**), thioperamide (**c**), and ciproxifan (**d**) and in sagittal monkey brain sections without blockade (**e**) or with 10 μM of unlabelled TASP0410457 (**f**) or ciproxifan (**g**). *Scale bar*: 10 mm

In vitro autoradiograms of rat brain sections with [^11^C]TASP0410457 illustrated the abundant specific binding in the striatum and neocortex (Fig. 7a). Radioactivity in the cerebellum and brainstem was low and not markedly altered by homologous or heterologous blockades of H_3_Rs (Fig. 7b–d). Regional autoradiographic labeling of monkey brain slices with [^11^C]TASP0410457 was consistent with the in vivo PET data (Fig. 7e). [^11^C]TASP0410457 binding in the monkey cerebellum and brainstem was noticeably decreased in the presence of 10 μM of unlabelled TASP0410457 or ciproxifan (Fig. 7f, g).

## Discussion

The present work provides a clear demonstration that a new PET radioligand with adequate binding and kinetic properties suitable for high-contrast and quantitative PET assays of central H_3_Rs can be generated from dihydroquinolinone derivatives. Screening of non-radiolabeled chemicals was carried out by the conjunctive use of information from in vitro assays and ex vivo LC-MS/MS measurements, resulting in the identification of two candidate compounds. Subsequent in vivo PET analyses of rat and monkey brains indicated the utility of these two ligands as imaging agents and revealed that the pyridinated compound [^11^C]TASP0410457 yielded a higher signal-to-background ratio, reflecting the abundance H_3_Rs, and good linearity of Lassen’s graphical plot across all examined brain regions, offering accurate quantitative measures of the receptor density at baseline and under occupancy by a drug with a sufficient dynamic range. Supported by these promising non-clinical items of evidence, [^11^C]TASP0410457 (a diminutive name, [^11^C]TASP457 is used in clinical works) has been applied to a human PET study and has been demonstrated to permit robust quantification of H_3_Rs in diverse brain regions (Kimura et al., manuscript submitted to Eur J Nucl Med Mol Imaging). These clinical data accordingly indicate the validity of our translational workflow for the identification of an appropriate H_3_R radioligand.

We employed ex vivo LC-MS/MS to select candidate compounds for PET imaging, because this technique has been successfully applied to label-free evaluations of potential PET ligands for opioid κ receptor and nociception/orphanin FQ receptor [16, 18]. Our present data also verified the capability of LC-MS/MS measurements in predicting in vivo performances of the H_3_R ligands when employed for PET examinations. Indeed, the relative order of ligand uptake in the rat forebrain determined by LC-MS/MS was overall in good agreement with the observations in rat PET. In light of the present and previous [16–20] demonstrations, LC-MS/MS quantifications of ex vivo samples can serve for capturing kinetic profiles of probable microdose imaging compounds with a reasonably high throughput. Meanwhile, a noticeable discrepancy between ex vivo and in vivo measures was also found in a small subset of data, including the peak cerebellar uptake of TASP0434988. In addition, ex vivo LC-MS/MS failed to determine concentrations of the compounds at 60 and 120 min after administration, as these values did not exceed the low limit of quantification of MS (0.15 ng/g tissue). These issues may be attributed to the sensitivity of the detection device and sample preparation for LC-MS/MS, which will need further improvements and optimizations.

It should also be considered that a radiometabolite of [^11^C]TASP0410457 in plasma gradually entered the CNS and raised PET signals in the brain while not affecting LC-MS/MS detection of the parent compound. We performed a preliminary analysis of metabolites in the rat brain after injection of non-radiolabeled TASP0410457 and TASP0434988 using an HPLC and ion trapping MS system and found that the sole detectable metabolite was generated by desmethylation of each parent compound at the ^11^C labeling site. Accordingly, it is likely that the major metabolic pathway leads to the production of a small, polar radiometabolite, such as [^11^C]formaldehyde, which is in accord with our HPLC analysis of plasma radiometabolites in monkeys. Moreover, our pilot assays in mice have suggested that this metabolite accounts for approximately 50 % of radioactivity in the brain at 30 min after intravenous injection of [^11^C]TASP0410457 (K. Kawamura, personal communication, 2015). In view of these factors potentially causing differences between LC-MS/MS and PET data, a practical workflow for the identification of the best PET ligand from an array of chemicals may be to select a few compounds exhibiting qualified kinetic properties in label-free ex vivo assays, followed by the determination of a radiolabeled ligand with the highest capability in rodent and nonhuman primate PET imaging, as conducted in the present work.

Our current results imply the applicability of [^11^C]TASP0410457 to investigations of H_3_Rs and evaluations of drugs acting on these receptors in the living human brain, which has been evidenced in clinical PET analyses (Kimura et al., manuscript submitted to Eur J Nucl Med Mol Imaging). Two radioligands, [^11^C]GSK189254 and [^11^C]MK-8278, have been non-clinically and clinically applied to quantify central H_3_Rs. Although [^11^C]GSK189254 exhibited sufficient brain uptakes, the kinetics of this radioligand exhibited a continuous increase over 90 min, hampering the estimation of regional *V*_T_ in the baboon and human striatum [11, 14, 29]. [^11^C]MK-8278 displayed more preferable kinetics (a rapid increase followed by a gradual decline in PET imaging time) than [^11^C]GSK189254, and but its time-stability data of *V*_T_ in the human brain showed a gradual increase in *V*_T_ over time [13]. The present study indicated that *V*_T_ of [^11^C]TASP0410457 in the monkey brain was relatively stable over time but increased by 7 % at maximum from 60 to 90 min, and this is presumably due to gradually accumulating radiometabolites in the brain but not stemming from slow kinetics of the radioligand binding. Hence, dynamic PET scans acquired for 60 min provide adequate quantitative measures of the radioligand kinetics in the monkey brain. As mentioned above, the major metabolite accounted for approximately 50 % of total radioactivity in the mouse brain at 30 min after radioligand injection (K. Kawamura, personal communication, 2015). In light of the finding that 86 and 53 % of total plasma activity in mice and monkeys, respectively, were derived from the radiometabolite at 30 min, the entry of the radiometabolite in the monkey brain should be less prominent than that in the mouse brain. Influences of the radiometabolite on *V*_T_ values in the human brain would be even less profound, because only 13 % of total plasma radioactivity at 30 min in humans arose from a metabolite identical to the molecule detected in animals (Kimura et al., manuscript submitted to Eur J Nucl Med Mol Imaging). Correspondingly, our clinical PET results showed smaller increase of *V*_T_ values along with extension of dynamic scan time from 60 to 90 min than the changes in a monkey (Kimura et al., manuscript submitted to Eur J Nucl Med Mol Imaging).

It is also noteworthy that pharmacokinetic evaluations of H_3_R ligands were quantitatively performable in rats using the cerebellum as a reference region lacking the target receptor. By contrast, the use of the cerebellum as a reference region to quantify H_3_R in the human and nonhuman primate brains remains to be revisited. A clinical PET study [^11^C]GSK189254 indicated that *V*_T_ values for this radioligand were markedly reduced in all ROIs including the cerebellum and pons by treatment with an H_3_R ligand, AZD5213, and it is accordingly likely that no reference region can be defined in the human brain [14]. However, PET imaging of the monkey and human brains with [^11^C]MK-8278 employed the pons as a reference region to estimate the occupancy of H_3_Rs by an antagonist/inverse agonist, MK-0249 [13, 30]. Our present data demonstrated the presence of displaceable binding sites for [^11^C]TASP0410457 and [^11^C]TASP0434988 in the monkey cerebellum and brainstem including the pons, similar to the previous reports on [^11^C]GSK189254 [11, 14, 29]. This finding was also consistent with the presence of specific binding components for H_3_R ligands in the monkey cerebellum demonstrated previously [30, 31]. Likewise, reference tissue models may not be available for describing the kinetics of [^11^C]TASP0410457 in the human brain, and thereby Lassen’s plot of *V*_T_ values estimated with an arterial input function will be required for clinical assays of the H_3_R occupancy by a drug.

## Conclusions

We developed a radioligand, [^11^C]TASP0410457, for a robust quantification of H_3_Rs in all brain regions and demonstrated the utility of ex vivo LC-MS/MS and in vivo PET assays for selecting appropriate imaging tracers from candidate compounds. In light of the current data, [^11^C]TASP0410457 will facilitate clarification of roles played by H_3_Rs in neuropsychiatric disorders and characterizations of emerging therapeutic agents acting on these receptors.

### Ethical approval

The animal experiments for ex vivo LC-MS/MS in rats were approved by the Animal Care Committee of Taisho Pharmaceutical Co., Ltd. All PET experiments using rats and monkeys were approved by the Committee for the Care and Use of Laboratory Animals of NIRS. All procedures performed in the studies were in accordance with the ethical standards of both institutions where the studies were conducted.

## Additional files

Additional file 1: Supplement methods and Table S1. Receptor binding selectivity of radioligand candidate compounds. (PDF 172 kb) Additional file 2: Figure S1. Analytical HPLC chromatogram of [^11^C]TASP0410457. (PDF 527 kb) Additional file 3: Figure S2. Analytical HPLC chromatogram of [^11^C]TASP0434988. (PDF 542 kb) Additional file 4: Figure S3. Analytical HPLC chromatogram of [^11^C]TASP0390136. (PDF 557 kb)
