# Supplementary material for: Development of TASP0410457 (TASP457), a novel dihydroquinolinone derivative as a PET radioligand for central histamine H3 receptors
Source: EJNMMI Res. 2016 Feb 9;6:11. doi: 10.1186/s13550-016-0170-2 (PMC4747952; doi:10.1186/s13550-016-0170-2)
Supplement: Additional file 1: Supplement methods and Table S1. — Receptor binding selectivity of radioligand candidate compounds. (PDF 172 kb) [file 13550_2016_170_MOESM1_ESM.pdf]

Supplemental Information for *EJNMMI Research*

Title:

Development of TASP0410457 (TASP457), a novel dihydroquinolinone derivative as a PET radioligand for central histamine H_3 receptors

Authors:

Kazumi Koga[1), 2), 3)], Jun Maeda[1)], Masaki Tokunaga[1)], Masayuki Hanyu[1)], Kazunori Kawamura[1)] Mari Ohmichi[2)], Toshio Nakamura[2)], Yuji Nagai[1)], Chie Seki[1)], Yasuyuki Kimura[1)], Takafumi Minamimoto[1)], Ming-Rong Zhang[1)], Toshimitsu Fukumura[1)], Tetsuya Suhara[1)], Makoto Higuchi[1)]

Affiliations:

1) Molecular Imaging Center, National Institute of Radiological Sciences
2) Taisho Pharmaceutical Co., Ltd.,
3) Tohoku University Graduate School of Medicine

Corresponding Author: Makoto Higuchi
Molecular Imaging Center, National Institute of Radiological Sciences
4-9-1 Anagawa, Inage-ku, Chiba, Chiba 263-8555, Japan
Phone: +81-43-206-4700 Fax: +81-43-253-0396
Email: mhiguchi@nirs.go.jp

Supplemental Methods

Chemical synthesis

^{1}H NMR spectra were obtained using JEOL JNM-ECA600 (JEOL Resonance Inc., Tokyo, Japan). Mass spectra were acquired on an Agilent 6150/Agilent 1290 Infinity (Agilent Technologies, Santa Clara, CA), and all MS instruments were operated in electrospray ionization (ESI) or atmospheric pressure chemical ionization (APCI) mode to detect positively charged ions.

Desmethyl precursor of TASP0410457

6-[(1-Cyclobutylpiperidin-4-yl)oxy]-1-(6-methoxypyridin-3-yl)-3,4-dihydroquinolin-2(1*H*)-one (21) (0.80 g) and 2M HCl in ethanol solution (2 mL) were stirred at 100°C for 16 h in a sealed tube. The reaction mixture was then cooled to room temperature and evaporated *in vacuo*, and the residue was purified using NH silica gel column chromatography (eluent: chloroform/methanol = 90/10) to yield a solid, which was further purified with recrystallization from methanol-water to produce the title compound (0.58 g) as a colorless powder (^{1}H NMR [600 MHz, CHLOROFORM-*d*] δ ppm 1.46 - 2.22 [m, 12 H] 2.60 [br. s., 2 H] 2.68 - 2.80 [m, 3 H] 2.92 - 3.05 [m, 2 H] 4.23 [br. s., 1 H] 6.48 [d, *J* = 9.08 Hz, 1 H] 6.62 - 6.69 [m, 2 H] 6.76 [d, *J* = 2.89 Hz, 1 H] 7.29 [dd, *J* = 9.50, 2.89 Hz, 1 H] 7.36 [d, *J* = 2.48 Hz, 1 H]; MS (ESI/APCI Dual] (Positive] m/z; 394(M+H)) .

Desmethyl precursor of TASP0434988

(i)

1-{4-[(tert-Butyldimethylsilyl)oxy]phenyl}-6-[(1-cyclobutylpiperidin-4-yl)oxy]-3,4-dihydroquinolin-2(1*H*)-one

6-(1-Cyclobutylpiperidin-4-yloxy)-3,4-dihydroquinolin-2(1*H*)-one (21) (1.0 g), (4-bromophenoxy) (tert-butyl)dimethylsilane (1.1 g), N,N'-dimethylethylenediamine (0.24 g), copper (I) iodide (0.13 g), and potassium bicarbonate (0.92 g) were stirred in toluene (5 mL) at 110°C for 4 h in a sealed tube. The reaction mixture was cooled to room temperature, poured into saturated ammonium chloride, and extracted with chloroform (3 times). The combined organic layers were dried over magnesium sulfate, evaporated *in vacuo*, and purified using NH silica gel column chromatography (eluent: hexane/ethyl acetate = 88/12~0/100) to yield the title compound (0.94 g) as a light yellow amorphous substance (^{1}H NMR [600 MHz, CHLOROFORM-*d*] δ ppm 0.23 [s, 6 H] 0.91 - 1.06 [m, 9 H] 1.60 - 2.21 [m, 12 H] 2.48 - 2.65 [m, 2 H] 2.67 - 2.82 [m, 3 H] 2.93 - 3.02 [m, 2 H] 4.15 - 4.25 [m, 1 H] 6.26 [d, *J* = 9.08 Hz, 1 H] 6.56 [dd, *J* = 8.88, 2.68 Hz, 1 H] 6.74 [d, *J* = 2.48 Hz, 1 H] 6.84 - 6.94 [m, 2 H] 7.03 - 7.11 [m, 2 H]; MS [ESI/APCI Dual] [Positive] m/z; 507[M+H]) .

(ii) 6-[(1-Cyclobutylpiperidin-4-yl)oxy]-1-(4-hydroxyphenyl)-3,4-dihydroquinolin-2(1*H*)-one (desmethyl precursor of TASP0434988)

To a stirred solution of 1-{4-[(tert-butyldimethylsilyl)oxy]phenyl}-6-[(1-cyclobutylpiperidin-4-yl)oxy]-3,4-dihydroquinolin-2(1*H*)-one (0.80 g) in tetrahydrofuran (4 mL), 1M tetrabutylammonium fluoride in tetrahydrofuran

(2.78 mL) was added at 0°C (ice bath), and stirred for 16 h at room temperature. The reaction mixture was evaporated *in vacuo*, and the residue was purified using NH silica gel column chromatography (eluent: hexane/ethyl acetate = 88/12~0/100) to yield a light-brown solid, which was further purified with recrystallization from ethanol to produce the title compound (0.28 g) as colorless powder (^{1}H NMR [600 MHz, CHLOROFORM-*d*] δ ppm 1.51 - 2.12 [m, 12 H] 2.44 - 2.57 [m, 2 H] 2.60 - 2.75 [m, 3 H] 2.86 - 2.98 [m, 2 H] 4.07 - 4.19 [m, 1 H] 6.22 [d, *J* = 8.67 Hz, 1 H] 6.49 [dd, *J* = 8.88, 2.68 Hz, 1 H] 6.67 [d, *J* = 2.89 Hz, 1 H] 6.73 - 6.79 [m, 2 H] 6.89 - 6.97 [m, 2 H]; MS [ESI/APCI Dual] [Positive] m/z; 393$[M+H]^{+}$) .

Desmethyl precursor of TASP0390136

(i)

(R)-1-{4-[(tert-Butyldimethylsilyl)oxy]phenyl}-6-[3-(2-methylpyrrolidin-1-yl)propoxy]-3,4-dihydroquinolin-2(1*H*)-one

(2R)-6-[3-(2-Methylpyrrolidin-1-yl)propoxy]-3,4-dihydroquinolin-2(1*H*)-one (21) (0.50 g), (4-bromophenoxy) (tert-butyl)dimethylsilane (0.99 g), N,N'-dimethylethylenediamine (0.20 g), copper (I) iodide (0.066 g) and potassium bicarbonate (0.48 g) were stirred in toluene (2 mL) at 110°C for 16 h in a sealed tube. The reaction mixture was cooled to room temperature, poured into saturated ammonium chloride, and extracted three times with chloroform. The combined organic layers were dried over magnesium sulfate, evaporated *in vacuo*, and purified using NH silica gel column

chromatography (eluent: hexane/ethyl acetate = 88/12 ~ 0/100) to produce the title compound (0.47 g) as a brown amorphous substance (^{1}H NMR [600 MHz, CHLOROFORM-*d*] δ ppm -0.03 [s, 6 H] 0.98 [s, 9 H] 1.05 [d, *J* = 5.78 Hz, 3 H] 1.35 [br. s., 2 H] 1.60 - 2.35 [m, 8 H] 2.70 - 2.79 [m, 2 H] 2.85 - 2.97 [m, 3 H] 3.94 [d, *J* = 5.37 Hz, 2 H] 6.24 – 6.28 [m, 1 H] 6.52 – 6.57 [m, 1H] 6.73 [d, *J* = 2.89 Hz, 1 H] 6.90 [d, *J* = 8.15 Hz, 2 H] 7.04 [d, *J* = 8.15 Hz, 2 H]; MS [ESI/APCI Dual] [Positive] m/z; 495$(M+H)^+$) .

(ii) (R)-1-(4-Hydroxyphenyl)-6-[3-(2-methylpyrrolidin-1-yl)propoxy]-3,4-dihydroquinolin-2(1*H*)-one (desmethyl precursor of TASP0390136)

To a stirred solution of (R)-1-{4-[(tert-Butyldimethylsilyl)oxy]phenyl}-6-[3-(2-methylpyrrolidin-1-yl)propoxy]-3,4-dihydroquinolin-2(1*H*)-one (0.47 g) in tetrahydrofuran (2 mL), 1M tetrabutylammonium fluoride in tetrahydrofuran (1.43 mL) was added at 0°C (ice bath), and stirred for 16 h at room temperature. The reaction mixture was evaporated *in vacuo*, and the residue was purified using NH silica gel column chromatography (eluent: chloroform/methanol = 100/0~90/10) to yield a solid, which was further purified using HPLC (column: Sunfire 30 × 50 mm [Waters, Milford, MA], eluent: 0.1% trifluoroacetic acid in acetonitrile/0.1% trifluoroacetic acid in water = 10/90~90/10). The resulting solid was washed with ethyl acetate and isopropyl ether to produce the title compound (0.17 g) as a colorless powder (^{1}H NMR [600 MHz, CHLOROFORM-*d*] δ ppm 0.98 [d, *J* = 5.78 Hz, 3 H] 1.32 [m,

2 H] 1.51 - 2.24 [m, 7 H] 2.69 [dd, J = 8.46, 6.40 Hz, 2 H] 2.82 - 2.92 [m, 3 H] 3.05 - 3.16 [m, 1 H] 3.86 [t, J = 6.40 Hz, 2 H] 6.20 [d, J = 9.08 Hz, 1 H] 6.45 [dd, J = 8.88, 2.68 Hz, 1 H] 6.63 - 6.69 [m, 3 H) 6.86 [d, J = 8.67 Hz, 2 H]; MS [ESI/APCI Dual) [Positive) m/z; 381$(M+H)^+$) .

Off-target binding of compounds

Sigma 1 receptor binding

The membrane preparations were made from Jurkat cell lines, and were incubated in sigma 1 binding buffer (0.1% bovine serum albumin in 50 mM Tris, pH7.4) containing 4 nM [^{3}H]pentazocine (PerkinElmer), and serial concentrations of the compound at 25°C for 2.5 h. The reaction was terminated by rapid filtration through a 96-well GF/C filter plate presoaked in 0.5% polyethyleneimine. The filters were washed with ice-cold 50 mM Tris (pH7.4) three times, dried, and filled with Microscint-o. The radioactivity retained on the filter was counted using a TopCount NXT. Nonspecific binding was determined as the amount of radioactivity under the above-mentioned conditions in the presence of 10 μM of haloperidol. The IC_{50} values were determined by nonlinear curve-fitting of the concentration-response data for each compound using Origin version 6.1087 (OriginLab Corporation, Northampton, MA).

Adrenergic α_{2c} receptor binding

Human α_{2c} receptor-expressing membrane preparations were purchased from PerkinElmer. The

membranes were incubated in binding buffer (1 mM EDTA and 0.1% bovine serum albumin in 50 mM Tris, pH7.4) containing 0.08 nM [^{3}H]MK-912 (PerkinElmer), and serial concentrations of the compound at 25°C for 1 h. The reaction was terminated by rapid filtration through a 96-well GF/C filter plate presoaked in 0.5% polyethyleneimine. The filters were washed with ice-cold wash buffer (50 mM Tris, pH7.4) five times, dried, and then filled with Microscint-o. The radioactivity retained on the filter was counted using a TopCount NXT. Nonspecific binding was determined as the amount of radioactivity under the conditions mentioned above in the presence of 10 μM of rauwolscine. The IC_{50} values were determined as for sigma 1 receptor binding.

M_4 receptor binding

Human M_4 receptor-expressing membrane preparations were purchased from PerkinElmer. The membranes were incubated in phosphate buffered saline (pH7.4) containing 0.4 nM [^{3}H]N-methyl-scopolamine (PerkinElmer) with or without 1 or 10 μM of compound at 27°C for 2 h. The reaction was terminated by rapid filtration through a 96-well GF/C filter plate presoaked in 0.5% polyethyleneimine. The filters were washed with ice-cold wash buffer (0.9% NaCl in 50 mM Tris, pH7.4) three times, dried, and then filled with Microscint-o. The radioactivity retained on the filter was counted using a TopCount NXT. Nonspecific binding was determined as the amount of radioactivity under the above-mentioned conditions in the presence of 1 μM of atropine.

Lipophilicity measurement

Log D was determined by partitioning each compound between an octanol and phosphate buffer (pH7.4) and determining the concentrations of TASP0410457, TASP0434988, and TASP0390136 in each layer using HPLC with ultraviolet (UV) detection.

Assays for P-glycoprotein-mediated transcellular transport of compounds

LLC-GA5-COL300 cells expressing human P-glycoprotein were seeded at a density of 4.5×10^5 cells/cm^2 onto polycarbonate membrane filters of 12-well cell culture insert plates and cultured at 37°C in a humidified incubator containing 5% CO_2 for 3 days. After each cell monolayer was pre-incubated at 37°C for 20 min in Hank's balanced salt solution containing 10 mM HEPES (HBSS buffer), transcellular transport experiments were initiated by adding the test compound solution to the apical or basal side of the cell culture inserts. HBSS buffer was sampled from the compartment opposite of that spiked with the test compounds at 0.5, 1, 1.5 and 2 h after initiation of the experiments, and concentrations of the test compounds were determined using LC-MS/MS. The apparent permeability coefficient (P_{app}) of the test compounds was calculated using the following equation:

$$P_{app} = dQ/dt \,/\, C_0 \,/\, A \qquad \text{Eq. 1,}$$

where dQ/dt represents the rate of permeation of the compounds, C_0 is the initial concentration of the test compounds (1 μM), and A is the area of the monolayer. The efflux ratio (ER) was defined as follows:

$$ER=P_{app,\ b\ to\ a}/P_{app,\ a\ to\ b} \quad \text{Eq. 2,}$$

where $P_{app,\ b\ to\ a}$ and $P_{app,\ a\ to\ b}$ represent P_{app} in the basal-to-apical (b to a) and apical-to-basal (a to b) directions, respectively.

Synthesis of [^{11}C]CH_3I

[^{11}C]CO_2 was produced using the $^{14}N(p,\alpha)^{11}C$ nuclear reaction in a 0.01% oxygen-containing nitrogen gas with 18-MeV proton beams. Following the bombardment process, [^{11}C]CO_2 was transferred to a vessel containing a 50-mM solution of lithium aluminum hydride in 300 μL of tetrahydrofuran at a temperature ranging from -10°C to -15°C. The resulting solution was concentrated to dryness, and a 57% solution of HI (300 μL) was added to the vessel. The mixture was then heated to 150°C to produce [^{11}C]CH_3I. [^{11}C]CH_3I was purified by passing through a column composed of Ascarite II and P_2O_5.

Radioligand synthesis

1-(4-[^{11}C]methoxyphenyl)-6-{3-[(2R)-2-methylpyrrolidin-1-yl]propoxy}-3,4-dihydroquinolin-2(1H)-one ([^{11}C]TASP0390136)

[^{11}C]CH_3I, the desmethyl precursor of TASP0390136 (1.0 mg), and 10 μL of 1 mM NaOH were mixed in 300 μL of dimethylformamide (DMF) at -15°C to -20°C, then heated at 100°C for 3 min (Fig. 1a); 1 mL of 50 mM phosphoric acid solution was then added to the reaction. The synthesized

[^{11}C]TASP0390136 was purified using HPLC (JASCO) on a SunFire C-18 column (10 × 250 mm, Waters, Milford, MA) with a mobile phase consisting of acetonitrile/50 mM phosphoric acid (25:75) at a flow rate of 4.0 mL/min. The eluent was monitored for its UV absorbance (254 nm) and radioactivity. The fraction corresponding to [^{11}C]TASP0390136, the retention time of which was approximately 9 min, was collected into a rotary evaporator flask containing polysorbate 80/EtOH (1:4; 100 μL). The solvent was removed *in vacuo* at 150°C, and the resulting residue was dissolved in 3 mL of sodium phosphate buffer (pH6.5).

6-[(1-Cyclobutylpiperidin-4-yl)oxy]-1-(6-[^{11}C]methoxypyridin-3-yl)-3,4-dihydroquinolin-2(1*H*)-one ([^{11}C]TASP0410457)

[^{11}C]CH_3I, the desmethyl precursor of TASP0410457 (0.8 mg), and cesium carbonate (8.0 mg) were mixed in 300 μL of DMF at -10°C to -15°C with N_2 gas, then heated at 100°C for 3 min (Fig. 1b). The synthesized [^{11}C]TASP0410457 was purified using the same process as that used for the synthesis of [^{11}C]TASP0390136, and a mobile phase consisting of acetonitrile/50 mM phosphoric acid (23:77) at a flow rate of 4.0 mL/min. The retention time of [^{11}C]TASP0410457 was approximately 8 min.

6-[(1-Cyclobutylpiperidin-4-yl)oxy]-1-(4-[^{11}C]methoxyphenyl)-3,4-dihydroquinolin-2(1*H*)-one ([^{11}C]TASP0434988)

[^{11}C]CH_3I, the desmethyl precursor of TASP0434988 (0.7 mg), and 10 μL of 1 mM NaOH were

mixed in 300 μL of DMF at -10°C to -15°C, then heated at 100°C for 3 min (Fig. 1c). The synthesized [^{11}C]TASP0434988 was purified using the same process as that used for the synthesis of [^{11}C]TASP0390136, and a mobile phase consisting of acetonitrile/50-mM phosphoric acid (24:76) at a flow rate of 5.0 mL/min. The retention time of [^{11}C]TASP0434988 was approximately 8 min.

Radioactivity of the chemicals was measured using a dose calibrator (IGC-3R Curiemeter; Aloka, Tokyo, Japan). Effluent radioactivity was measured using a NaI(TI) scintillation detector system (Ohyo Koken Kogyo Co., Ltd, Tokyo).

Measurements of radiochemical purity and specific activity of radioligands

Synthesized [^{11}C]TASP0410457 was analyzed by HPLC on a SunFire C-18 column (4.6 × 250 mm, Waters) with a mobile phase consisting of acetonitrile/50 mM phosphoric acid (21:79) at a flow rate of 1 mL/min. A representative chromatogram of [^{11}C]TASP0410457 is shown in Supplemental Fig. 1.

Synthesized [^{11}C]TASP0434988 was analyzed using HPLC performed on a SunFire C-18 column (4.6 × 250 mm, Waters) with a mobile phase consisting of acetonitrile/50 mM phosphoric acid (24:76) at a flow rate of 1 mL/min. A representative chromatogram of [^{11}C]TASP0434988 is shown in Supplemental Fig. 2.

Synthesized [^{11}C]TASP0390136 was analyzed using HPLC performed on a SunFire C-18 column (4.6 × 250 mm, Waters) with a mobile phase consisting of acetonitrile/50 mM phosphoric acid

(25:75) at a flow rate of 1 mL/min. A representative chromatogram of [^{11}C]TASP0390136 is shown in Supplemental Fig. 3.

Autoradiography with [^{11}C]TASP0410457

Twenty-micrometer-thick sagittal rat brain sections were pre-incubated with autoradiography buffer (50 mM Tris containing 120 mM NaCl, 2 mM KCl, 1 mM $MgCl_2$, and 1 mM $CaCl_2$, pH7.4) for 30 min, then incubated in the autoradiography buffer containing 5 nM [^{11}C]TASP0410457 at 25°C for 1 h. The sections were washed two times with ice-cold autoradiography buffer for 5 min, rinsed with ice-cold water for 10 s, and then quickly dried. The radioactivity retained on the sections was determined using a BAS imaging plate and the BAS5000 system (GE Healthcare, Piscataway, NJ). For a blocking study, brain slices were reacted with [^{11}C]TASP0410457 in the presence of 10 μM of TASP0410457, thioperamide, or ciproxifan.

Similarly, autoradiography using 20-μm-thick sagittal rhesus monkey brain sections was conducted. For a blocking study, the brain slices were reacted with [^{11}C]TASP0410457 in the presence of 10 μM of TASP0410457 or ciproxifan.

Supplemental Table 1

Off-target binding of compounds assessed using an in-house assay (M_4) and a commercial battery (all others) (Cerep, Celle L'Evescault, France).

		TASP0410457		TASP0434988		TASP0390136	
		Concentration	*% Inhibition*	*Concentration*	*% Inhibition*	*Concentration*	*% Inhibition*
Adenosine	A1	10 µM	-13	1 µM	3	10 µM	-1
Adrenaline	alpha 1A	10 µM	35	1 µM	14	10 µM	32
	alpha 1B	10 µM	8	1 µM	6	10 µM	19
	alpha 1D	10 µM	29	1 µM	25	10 µM	23
	alpha 2B	10 µM	28	1 µM	-8	1 µM	-16
	beta 1	10 µM	4	1 µM	3	10 µM	-3
	beta 2	10 µM	0	1 µM	2	10 µM	-9
	beta 3	10 µM	0	1 µM	-22	10 µM	-49
	Norepinephrine transporter	10 µM	-1	1 µM	1	10 µM	1
Benzodiazepine (central)		10 µM	-4	1 µM	-2	10 µM	-19
Benzodiazepine (peripheral)		10 µM	-8	1 µM	-9	10 µM	-6
Bradykinin	B1	10 µM	5	1 µM	-10	10 µM	0
Ca2+ channel (L, dihydropyridine site)		10 µM	-23	1 µM	-27	10 µM	20
Ca2+ channel (N)		10 µM	-10	1 µM	-6	10 µM	10
Cannabinoid	CB1	10 µM	-19	1 µM	2	10 µM	-15
	CB2	10 µM	-10	1 µM	-8	10 µM	-23
Cholecystokinin	CCK1 (CCKA)	10 µM	-1	1 µM	6	10 µM	7
Dopamine	D1	10 µM	-4	1 µM	-2	10 µM	7
	D2L	10 µM	-26	1 µM	-8	10 µM	10
	D3	10 µM	19	1 µM	7	10 µM	14
	D4.4	10 µM	-5	1 µM	6	10 µM	-12
	D5	10 µM	5	1 µM	1	10 µM	-41
GABA	A1 (alpha 1,beta 2,gamma 2)	10 µM	-13	1 µM	-9	10 µM	-7
	B (1b)	10 µM	-15	1 µM	-17	10 µM	0
Galanin	GAL1	10 µM	-9	1 µM	-5	10 µM	-19
Glucocorticoid	GR	10 µM	6	1 µM	-3	10 µM	-15
Glutamate	AMPA[*1]	10 µM	1	1 µM	0	10 µM	-18
	Kainate	10 µM	-12	1 µM	4	10 µM	-11
	NMDA[*2]	10 µM	11	1 µM	-7	10 µM	-2

	Glycine[*3]	10 µM	-5	1 µM	-8	10 µM	12
	PCP[*4]	10 µM	-2	1 µM	9	10 µM	-2
Histamine	H1	10 µM	6	1 µM	-9	1 µM	5
	H2	10 µM	-16	1 µM	13	10 µM	-7
	H4	10 µM	-4	1 µM	-7	10 µM	2
Imidazoline	I2	10 µM	1	1 µM	16	10 µM	23
K+ channel	KATP[*5]	10 µM	5	1 µM	-4	10 µM	22
	KV[*6]	10 µM	-11	1 µM	-1	10 µM	-15
	SKCa[*7]	10 µM	-1	1 µM	-2	10 µM	7
Melanocortin	MC1	10 µM	2	1 µM	0	10 µM	-10
Muscarinic	M1	10 µM	7	1 µM	-10	10 µM	11
	M2	10 µM	22	1 µM	-16	10 µM	37
	M3	1 µM	12	1 µM	13	10 µM	30
	M4	10 µM	19	1 µM	23	1 µM	13
	M5	10 µM	30	1 µM	8	10 µM	11
Nicotinic	Neuronal alpha 4 beta 2	10 µM	17	1 µM	7	10 µM	13
	Neuronal alpha 7	1 µM	41	1 µM	37	10 µM	7
Neurokinin	NK1	10 µM	-19	1 µM	-18	10 µM	-9
	NK2	10 µM	3	1 µM	-7	10 µM	6
	NK3	10 µM	-4	1 µM	-15	10 µM	12
Neuropeptide Y1		10 µM	1	1 µM	-5	10 µM	-16
Opioid	delta 2 (DOP)	10 µM	-1	1 µM	0	10 µM	3
	kappa (KOP)	10 µM	-6	1 µM	-12	10 µM	11
	mu (MOP)	10 µM	8	1 µM	-15	10 µM	10
Serotonin	5-HT1A[*8]	10 µM	-5	1 µM	-4	10 µM	2
	5-HT1B	10 µM	25	1 µM	3	10 µM	-25
	5-HT1D	10 µM	17	1 µM	-12	10 µM	5
	5-HT2A	10 µM	7	1 µM	-1	10 µM	9
	5-HT2B	10 µM	17	1 µM	-20	10 µM	-13
	5-HT2C	10 µM	-15	1 µM	-3	10 µM	6
	5-HT3	10 µM	9	1 µM	-3	10 µM	-3
	5-HT4e	10 µM	-3	1 µM	4	10 µM	20
	5-HT5a	10 µM	-5	1 µM	-18	10 µM	-8
	5-HT6	10 µM	-1	1 µM	-2	10 µM	-3
	5-HT7	10 µM	-5	1 µM	-2	10 µM	2
	5-HT transporter	10 µM	17	1 µM	7	10 µM	12
Somatostatin	sst1	10 µM	3	1 µM	4	10 µM	3
Vasopressin	V1a	10 µM	0	1 µM	9	10 µM	13

[*1]α-amino-3-hydroxy-5-methyl-4-isoxazolepropionic acid receptor; [*2]N-methyl-D-aspartate receptor; [*3]Strychnine-insensitive glycine-binding module of NMDA receptor; [*4]Phencyclidine-binding site of NMDA receptor; [*5]ATP-sensitive potassium channel; [*6]Voltage-dependent potassium channel; [*7]small conductance calcium-activated potassium channel; [*8]5-hydroxytryptamine 1A receptor
