# Supplementary figures and images for: Development of TASP0410457 (TASP457), a novel dihydroquinolinone derivative as a PET radioligand for central histamine H3 receptors
Source: EJNMMI Res. 2016 Feb 9;6:11. doi: 10.1186/s13550-016-0170-2 (PMC4747952; doi:10.1186/s13550-016-0170-2)

Supplemental Fig. 1

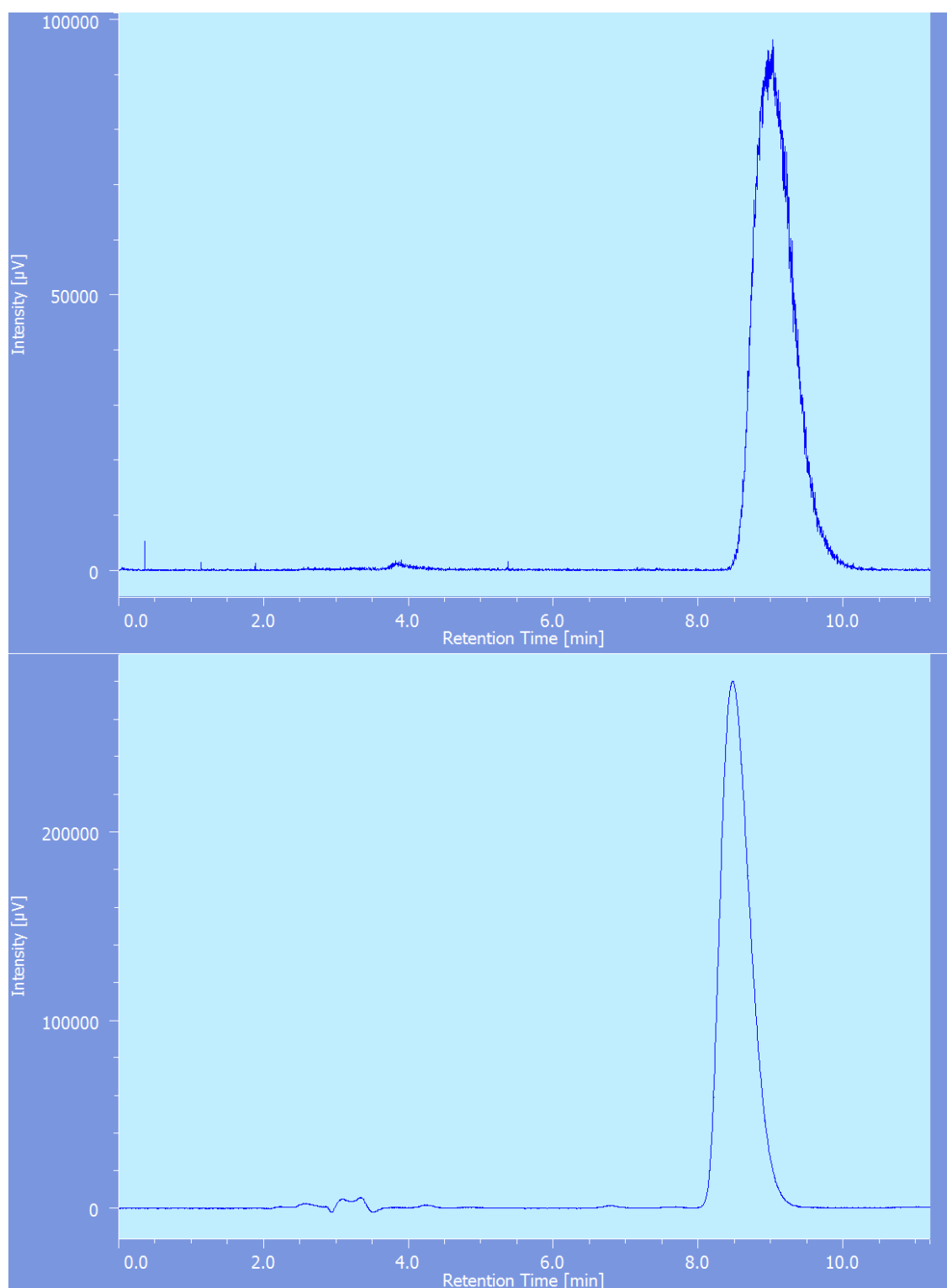

Supplement: Additional file 2: Figure S1. — Analytical HPLC chromatogram of [11C]TASP0410457. (PDF 527 kb) [file 13550_2016_170_MOESM2_ESM.pdf]

Supplemental Fig. 2

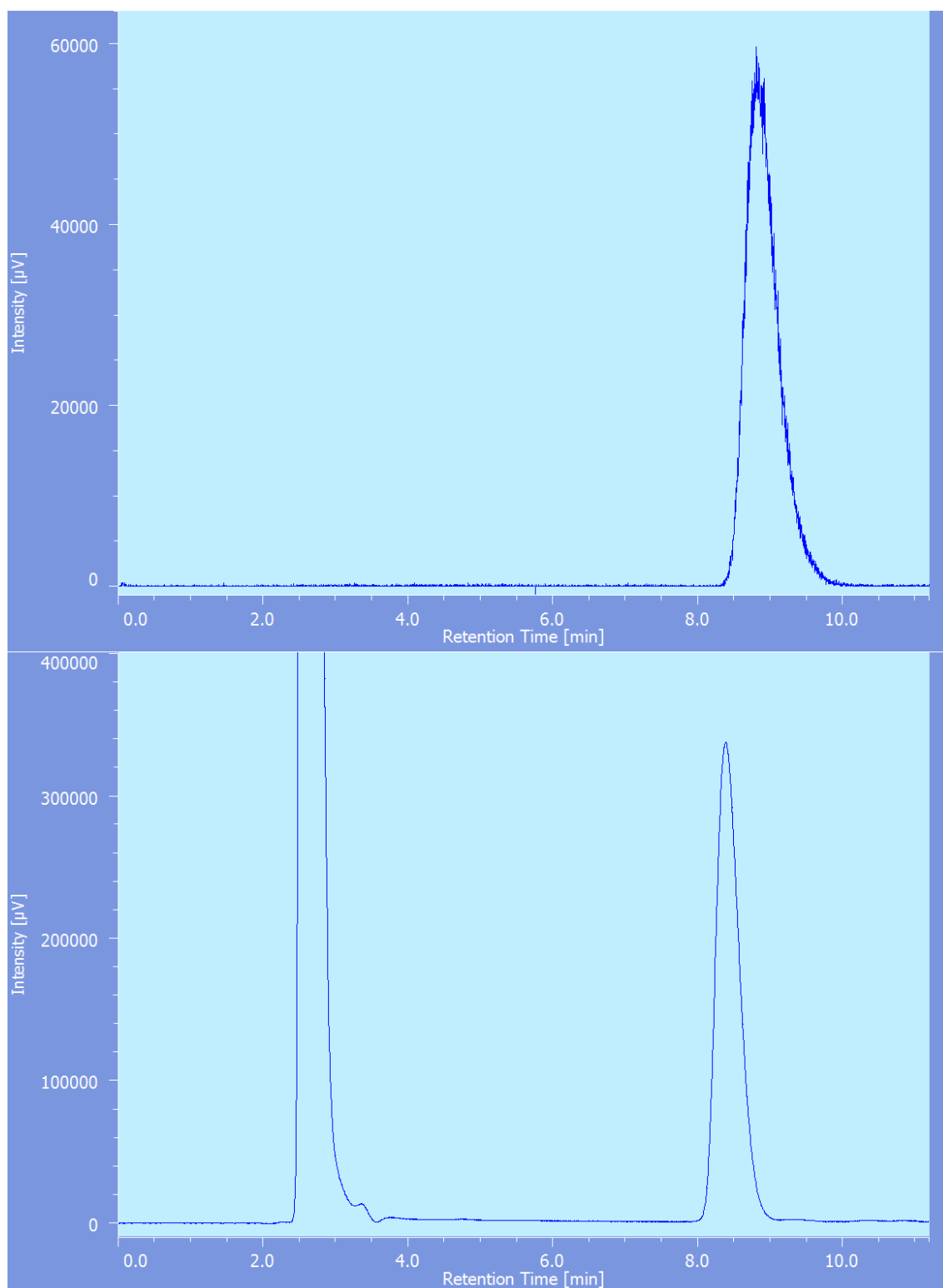

Supplement: Additional file 3: Figure S2. — Analytical HPLC chromatogram of [11C]TASP0434988. (PDF 542 kb) [file 13550_2016_170_MOESM3_ESM.pdf]

Supplemental Fig. 3

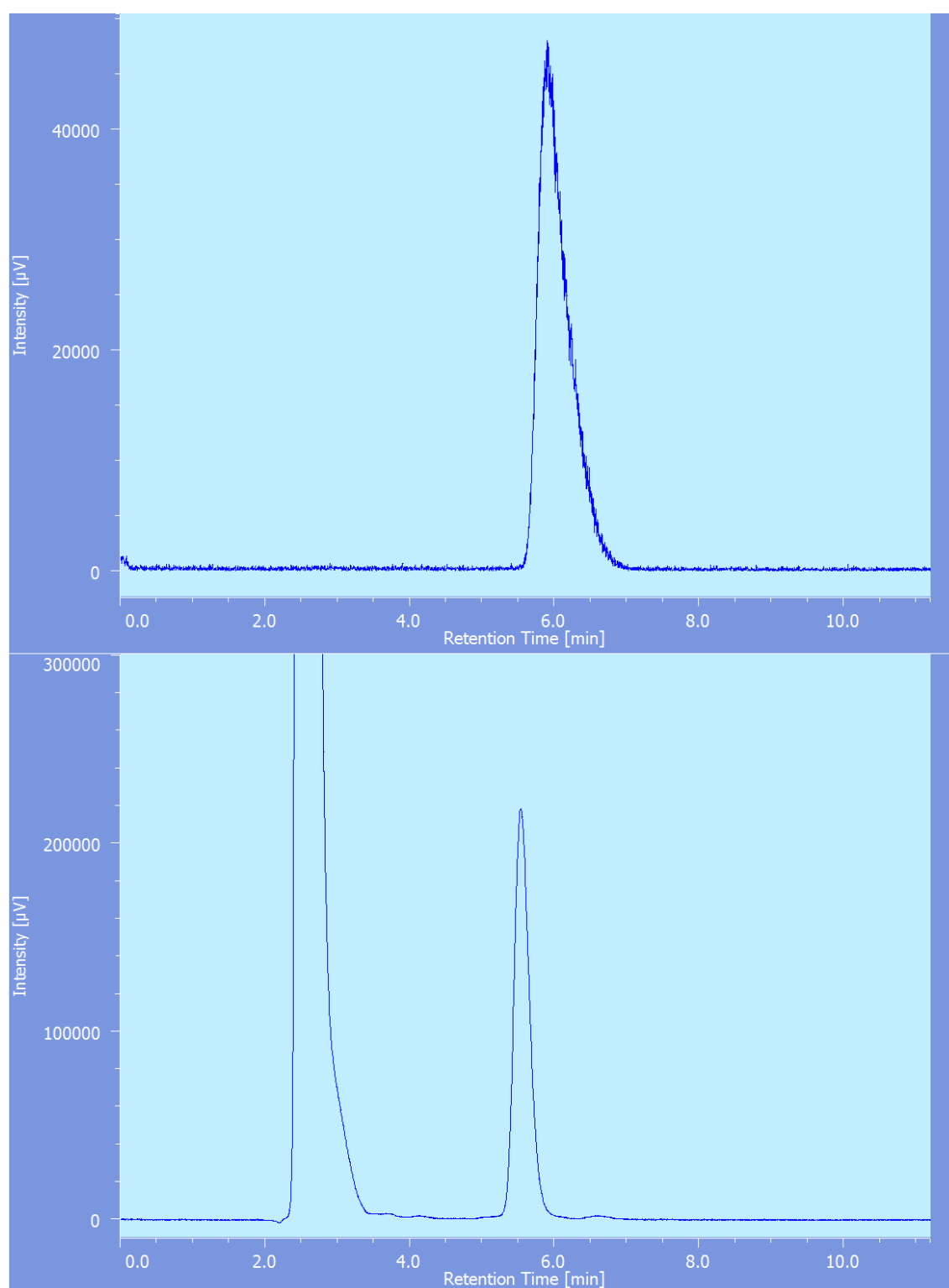

Supplement: Additional file 4: Figure S3. — Analytical HPLC chromatogram of [11C]TASP0390136. (PDF 557 kb) [file 13550_2016_170_MOESM4_ESM.pdf]
